# Une cause inhabituelle de dysphagie haute: schwannome de la l oge hyo-thyro-épiglottique

**DOI:** 10.11604/pamj.2017.28.295.14146

**Published:** 2017-12-06

**Authors:** Jalila Ben Ayad, Chaimae Nekro, Safaa Rokhssi, Omar Lassikri, Mohamed Anass Benbouzid, Leila Essakalli

**Affiliations:** 1 Service d’Oto-Rhino-Laryngologie et Chirurgie Cervico-Faciale, Hôpital des Spécialités, CHU Ibn Sina, Rabat, Maroc

**Keywords:** Schwanomme, loge hyo-thyro-épiglottique, chirurgie, Schwanomma, pre-epiglottic space, surgery

## Abstract

Les schwannomes sont des tumeurs mésenchymateuses, bien encapsulées des nerfs périphériques, de croissance lente. La localisation laryngée est exceptionnelle, nous présentons un cas non décrit dans la littérature de shwannome localisé au niveau de la loge hyothyroépiglottique. Une patiente de 50 ans, qui présentait une sensation de corps étranger depuis 4 ans, évoluant vers une dysphagie haute associée à une voix rauque. La laryngoscopie directe en suspension objectivait une masse supraglottique sous muqueuse régulière, réduisant la lumière pharyngo-laryngée. La TDM et l'IRM concluaient à une tumeur bénigne de la loge hyo-thyro-épiglottique. Une exérèse chirurgicale par voie externe a été réalisée et une trachéotomie première était nécessaire. L'examen histologique avec une étude immunohistochimique confirmait un schwannome bénin. Les suites post opératoires étaient simples. Aucune récidive n'était objectivée après 2 ans de recul.

## Abstract (en)

Schwannomas are well encapsulated mesenchymal tumors of peripheral nerves, with slow growth. The laryngeal schwanomma is exceptional, we present a case not described in the literature of shwannoma in the pre epiglottic space. A 50-year-old woman, who had a history of foreign body sensation for 4 years ago, progressing to high dysphagia and hoarseness. Direct laryngoscopy in suspension demonstrated a regular submucosal mass in the supraglottic space, reducing pharyngo-laryngeal space. CT and MRI concluded with a benign tumor of the preepiglottic space. External surgical excision was performed and a primary tracheotomy was required. The histological examination with an immunochemical study confirmed a benign schwannoma. There was no complication postoperatively. The outcome was excellent after 2 years of follow-up.

## Introduction

Le schwannome ou neurinome est une tumeur mésenchymateuse bénigne, bien encapsulée, qui nait des cellules de la gaine de Schwann et de l'épinèvre entourant le nerf périphérique [1]. La localisation laryngée est exceptionnelle et lorsqu'elle existe les sites laryngés fréquemment concernés sont le repli ary-épiglottique, l'aryténoide et la bande ventriculaire [2,3]. Nous rapportons le premier cas d'un schwannome laryngé localisé au niveau le l'espace pré-épiglottique.

## Patient et observation

**Figure 1:**
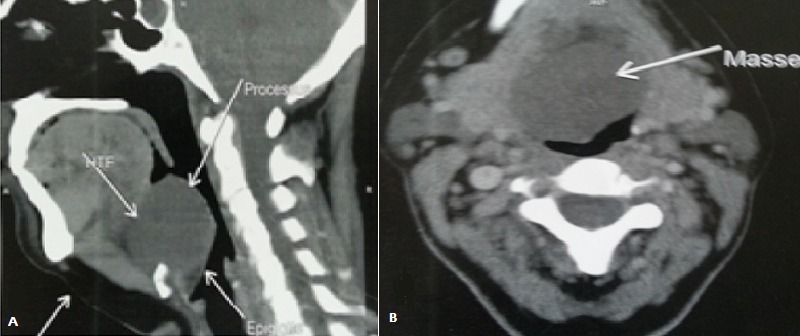
TDM cervicale en coupe sagittale (A) et axiale (B) montrant un processus lésionnel hypodense de la loge hyothyroépiglottique

**Figure 2:**
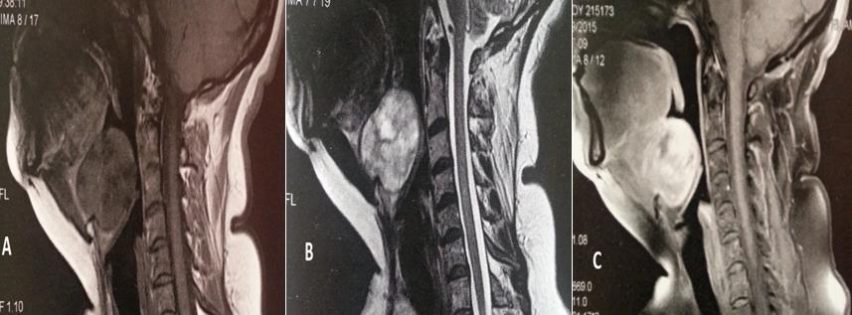
IRM cervicale en coupe sagittale en séquences pondérées T1 (A), T2 (B) et après ingection de gadolinium (C), montrant le schwanomme de la loge hyothyroépiglottique

**Figure 3:**
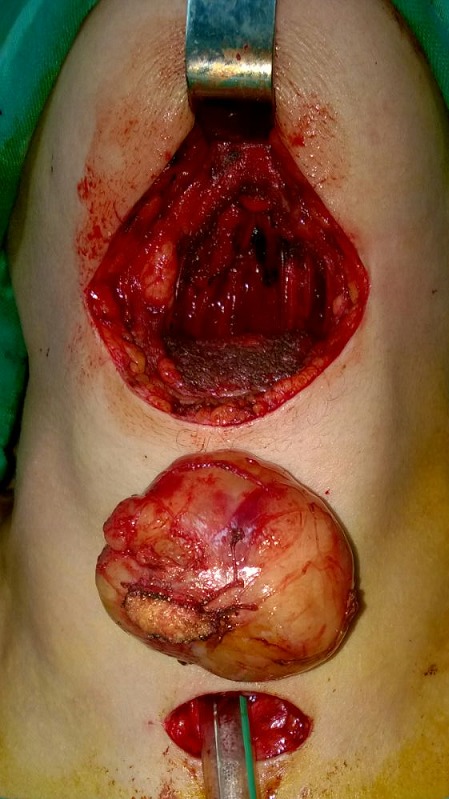
Image de schwanomme extra vivo après exérèse par cervicotomie médiane sushyoidienne avec intubation trachéale

Une patiente de 50 ans, aux antécédents de tuberculose pleurale traitée il y a 26 ans, qui présentait depuis 4 ans une sensation de corps étranger laryngé évoluant vers une dysphagie haute progressive aux solides depuis 1 an, associée à une voix rauque. L'examen cervical était normal. La laryngoscopie directe retrouvait une masse sous muqueuse supraglottique, bombant les vallécules et réduisant la lumière pharyngo-laryngée à 70%, le plan glottique n'était pas visible et les deux sinus piriformes étaient d'aspect sain. La TDM a objectivé un processus lésionnel de la loge hyo-thyro-épiglottique, de densité tissulaire régulière non vasculaire, mesurant 44,5 x 35,3 x 41 mm de diamètres, sans signe d'agressivité locale (Figure 1). L'IRM retrouvait un processus tissulaire sous muqueux en hyposignal T1, en hypersignal T2 et se rehaussant de façon hétérogène et intense après injection de gadolinium (Figure 2). Une tumeur bénigne fut suspectée et aucune biopsie n'a été réalisée. La tumeur étant obstructive, une trachéotomie première était nécessaire avant l'exérèse tumorale pour l'intubation. Celle-ci a été abordée par une cervicotomie sushyoïdienne, qui a permis une énucléation complète de la tumeur (Figure 3). L'orifice de trachéotomie était fermé en fin d'intervention. L'analyse anatomopathologique avec une étude immunohistochimique concluait à un schwannome bénin. Les suites post-opératoires étaient excellentes. L'alimentation orale était reprise à J1 post opératoire. Après 18 de recul, la nasofibroscopie objective une muqueuse pharyngo-laryngée saine avec une mobilité des cordes vocales conservée.

## Discussion

La loge hyo-thyro-époglottique est un espace endolaryngé large, constitué principalement de tissu adipeux et fibro-élastique lâche. Les tumeurs bénignes de cette région anatomique sont très rares. Deux types histologiques ont été décrits notamment l'hamartome et le choristome provenant du tractus thyréogloss [4]. Il existe principalement deux types de tumeurs bénignes neurogènes du larynx : le schwannome et le neurofibrome et l'ensemble représente 0,1% à 1,5% des tumeurs bénignes du larynx [1]. Les schwannomes sont des tumeurs solitaires et bien encapsulés, au contraire des neurofibromes qui apparaissent non encapsulé avec des limites irrégulières et s'associent fréquemment à un tableau diffus de neurofibromatoses [1]. Le schwannome laryngé provient de la branche interne du nerf laryngé après sa pénétration dans la membrane hyo-thyroïdien [5]. Il se développe principalement au niveau de l'espace supraglottique sous forme d'une masse sous muqueuse régulière, les sites fréquemment concernés sont le repli aryépiglottique, le cartilage aryténoïde et la bande ventriculaire [3,5]. Des rares cas de schwannome de l'étage sous glottique sont décrits [3]. Cette lésion peut toucher tous les âges, mais son pic d'incidence se situe entre la 2^ème^ et la 5^ème^ décennie [5]. Il n'y a pas de prédilection de sexe, Cependant certains auteurs rapportent une prédominance féminine [2]. Les schwannomes ont une lente évolution, pouvant rester asymptomatique pendant plusieurs années. La présentation clinique est liée à la localisation de la tumeur et son volume, les symptômes les plus fréquents sont la sensation de corps étranger, l'odynophagie, l'enroument de la voix, la dysphagie, la dyspnée et le stridor [2,5]. Un cas de décès lié à une forme asphyxiante de schwannome laryngé a été rapporté [6].

L'examen cervical de notre patiente était normal, cependant elle existe des formes cliniques avec une extension extralaryngée pouvant mimer une laryngocèle [7]. La TDM et IRM jouent un rôle primordial pour déterminer la localisation exacte de la tumeur et son extension, cependant la confirmation diagnostique reste histologique. Trois caractères histologiques déterminent le diagnostic : la présence de la capsule, la présence des cellules d'Antoni et la positivité à S100 [8]. Le traitement de référence des shwannomes laryngés est chirurgical. L'abord chirurgicale dépend de la taille et de la localisation de la tumeur, ainsi la microchirurgie endolaryngée au laser ou instrumentale est indiquée pour les schwannomes de petite taille et la chirurgie par voie externe pour les tumeurs les plus volumineuses [9]. La chirurgie transorale robot-assistée est autre modalité thérapeutique récemment décrite pour l'exérèse des shwannomes laryngés supraglottique [10]. Différentes approches chirurgicales ont été décrites pour abordées les tumeurs les plus volumineuses telles qu'une thyrotomie médiane ou latérale et une pharyngotomie latérale [3,5]. La voie transhyoidienne était nécessaire chez notre patiente pour aborder cette localisation exceptionnelle de schwannome. Le pronostic des schwanomes laryngés est excellent, des rares cas de récidives ont été rapportés et qui sont surtout liées à une exérèse incomplète [2].

## Conclusion

Le schwannome laryngé est très rare surtout sa localisation au niveau de l'espace pré-épiglottique. Il faut savoir l'évoquer devant toute masse sous muqueuse supraglottique. La TDM et L'IRM sont primordiales pour le diagnostic et le bilan d'extension. La localisation et la taille de la tumeur déterminent la voix d'abord chirurgical. Le pronostic est excellent sous réserve d'une exérèse tumorale complète.

## Conflits d’intérêts

Les auteurs ne déclarent aucun conflit d'intérêts.
